# Identification of Polycystic Ovary Syndrome (PCOS) Specific Genes in Cumulus and Mural Granulosa Cells

**DOI:** 10.1371/journal.pone.0168875

**Published:** 2016-12-20

**Authors:** Alp Aydos, Aykut Gurel, Yasemin Oztemur Islakoglu, Senem Noyan, Bagdagul Gokce, Tolga Ecemis, Cemil Kaya, Arif Tarik Aksu, Bala Gur Dedeoglu

**Affiliations:** 1 Biotechnology Institute, Ankara University, Ankara, Turkey; 2 Test Tube Babies Unit, HRS Women Hospital, Ankara, Turkey; 3 Test Tube Babies Unit, Medicana International Ankara Hospital, Ankara, Turkey; 4 Department of Gynecology and Obstetrics, Liv Hospital, Ankara, Turkey; 5 Department of Gynecology and Obstetrics, TOBB ETU Hospital, Ankara, Turkey; 6 Department of Gynecology and Obstetrics, HRS Women Hospital, Ankara, Turkey; John Hopkins University School of Medicine, UNITED STATES

## Abstract

Polycystic ovary syndrome (PCOS) is a metabolic and endocrine disorder which affects women of reproductive age with prevalence of 8–18%. The oocyte within the follicle is surrounded by cumulus cells (CCs), which connect with mural granulosa cells (MGCs) that are responsible for secreting steroid hormones. The main aim of this study is comparing gene expression profiles of MGCs and CCs in PCOS and control samples to identify PCOS-specific differentially expressed genes (DEGs). In this study, two microarray databases were searched for mRNA expression microarray studies performed with CCs and MGCs obtained from PCOS patients and control samples. Three independent studies were selected to be integrated with naive meta-analysis since raw meta-data from these studies were found to be highly correlated. DEGs in these somatic cells were identified for PCOS and control groups. This study enabled us to reveal dysregulation in MAPK (mitogen activated protein kinase), insulin and Wnt signaling pathways between CCs and MGCs in PCOS. The meta-analysis results together with qRT-PCR validations provide evidence that molecular signaling is dysregulated through MGCs and CCs in PCOS, which is important for follicle and oocyte maturation and may contribute to the pathogenesis of the syndrome.

## Introduction

Polycystic ovary syndrome (PCOS) is the most common endocrine disorder affecting women of reproductive age [1]. The syndrome has heterogeneous clinical characteristics, including hyperandrogenemia, ovulatory dysfunction, polycystic ovarian morphology (PCOM) and metabolic disorders (obesity, insulin resistance and diabetes). Additionally, it is a common cause of anovulation and female infertility through impairing oocyte-follicle maturation [2].

Mural granulosa cells (MGCs) and cumulus cells (CCs), which are the somatic cells that make up the oocyte microenvironment, secrete steroid hormones and produce growth hormone involved in oocyte development. One of the basic functions of MGCs is arresting the oocyte in the meiotic phase until ovulation by secreting oocyte maturation inhibitor (OMI) [3]. At the beginning of the menstrual cycle, MGCs receive external hormone signals to resume oocyte maturation. This activation signal is then transmitted to the CCs, which are in direct contact with the oocyte. This cumulus-oocyte complex (COC) has gap junctions for establishing a network linking CCs and oocyte. The CCs supply the oocyte with pyruvate needed to meet its energy requirements, and supply nucleotides and amino acids [4].

Microarray technology allows gene expression profiles to be identified, which is important for understanding and revealing the molecular mechanisms of diseases. However, only one study has used this method to compare MGC and CC gene expression profiles in control samples [5]. Assembling microarray datasets from different sources, known as meta-analysis, provides an extended perspective with improved statistical power due to increased sample size by including robust, replicable and accurate information from separate data sources [6]. In this study, our meta-analysis combined expression data for MGCs and CCs obtained from both PCOS and control samples. This allowed us to explore PCOS-specific differentially expressed genes (DEGs) between MGCs and CCs. Comparing the gene expression profiles of MGCs and CCs helped us to identify the important specific functions of these two somatic cell types in PCOS. The aim of the study was to compare gene expression profile data provided from the Gene Expression Omnibus [7] (GEO, www.ncbi.nlm.nih.gov/geo/) and ArrayExpress [8] (www.ebi.ac.uk/arrayexpress) databases, comparing MGCs and CCs in PCOS without differentially expressed genes in women with normal ovarian function.

## Materials and Methods

**Fig 1:**
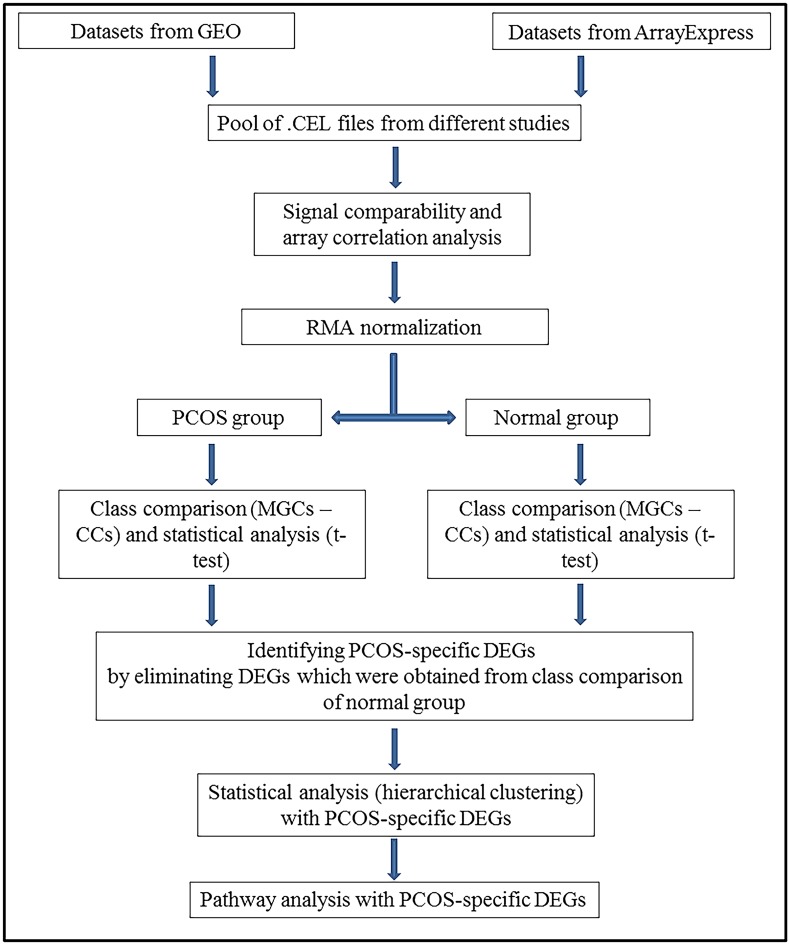
Flowchart of the process for identifying PCOS-specific differentially expressed genes (DEGs) and related pathways.

The main flowchart of the study is given in Fig 1.

### Microarray studies extracted from databases and *in-silico* subjects

Expression data of MGCs and CCs for PCOS patients and controls were searched for in the GEO and ArrayExpress databases. We used “cumulus cells, granulosa cells, PCOS” key words and their combinations. Database interfaces were filtered by “Organism: Human” and “Platform: Affymetrix GeneChip HG-U133 Plus 2.0 (Platform ID: GPL570 for GEO, A-AFFY-44 for ArrayExpress)”. The expression data were chosen from studies performed using the same microarray platform (GeneChip HG-U133 Plus 2.0 (Affymetrix Inc., Santa Clara, CA)) to eliminate platform based variabilities. Using these criteria, we chose three datasets for the meta-analysis: two with GEO accession numbers GSE34526 [9], GSE10946 [10] and one ArrayExpress accession number E-MEXP-3641 [5].

### Experimental subjects

MGCs and CCs were obtained from a total sample of 12 PCOS patients diagnosed according to the 2003 Rotterdam revised criteria during oocyte pick up for *in vitro* fertilization. The IVF protocol was as follows. In all patients, controlled ovarian hyperstimulation was performed using a single dose of 150 IU gonadotropin ß on cycle day 2 or 3. To prevent premature ovulation, all patients received the antagonist from the sixth stimulation day before ovulation was induced 10 days after stimulation using 5,000 IU hCG. In case of risk of overhyperstimulation syndrome, 0.2 mg GnRH-agonist was used. Follicular puncture was performed exactly 35 hours after induction of ovulation [11].

This study and the use of the tissue material in the project were approved by the Research Ethics Committee of Ankara University School of Medicine. Written consent was obtained from all patients in accordance with the Helsinki Declaration.

#### Isolation of MGCs and CCs from follicular fluid

MGCs and CCs were collected from follicular fluid (FF) during oocyte pick-up (OPU) 36 hours after hCG injection. Cumulus-oocyte complexes (COCs) were taken to culture medium for IVF procedure. The remaining follicular fluid from the same patient was pooled for MGC extraction. Oocytes were extracted by dissection from the COC with the remaining cumulus cells being pooled and collected for our study. Pooled CCs and FF were transferred to our laboratory at 4°C within an hour to prevent cell death. After transfer, MGCs were isolated from FF using Biocoll phase-gradient separating solution (Biochrom AG) at 600 g for 15 min at 4°C. The interphase layers, which were granulosa cells, were collected and washed twice with 1X Phosphate Buffered Saline (PBS) (Lonza).

After GCs extraction, total RNA was extracted from cumulus and granulosa cells using TRIzol Reagent (Life Technologies, Inc.). The concentration and purity of the extracted total RNA was assessed using an ND-1000 Nanodrop spectrophotometer (NanoDrop) before storage at -80°C until further analysis. Finally, complementary DNA (cDNA) was synthesized using 500 ng total RNA with Transcriptor Reverse Transcriptase (Roche), according to the manufacturer’s instructions.

### Signal comparability and array correlation analysis for combining datasets

Each dataset obtained from the databases was uploaded to ArrayAnalysis [12] (http://www.arrayanalysis.org/) for array correlation and signal-array comparability analysis. Highly correlated (Pearson’s r≥0.8) array datasets were selected after array correlation analysis.

### Microarray data normalization and rearrangement of the meta-data

Since the normalized data obtained from the three independent microarray studies were found to be highly correlated with each other, all three datasets were combined and normalized as a single dataset by Robust Multi-Array Average (RMA) method using BRB-ArrayTools (v4.4.1) [13]. Since this combination protocol is known as naive meta-analysis [14], so we refer to the data generated from the meta-analysis as meta-data.

### Microarray data analysis

After normalization of the meta-data, two classes were defined for identifying DEGs between CCs and MGCs for PCOS and control groups. A class comparison (unpaired t-test with random variance model [15]) was performed with minimum 4 fold-change (p≤0.001) for each PCOS and control group.

DEGs between MGC and CC in the control group were differentiated from those in the PCOS group using VENNY (http://bioinfogp.cnb.csic.es/tools/venny/index.html.) [16] to identify those genes expressed only in PCOS (PCOS-specific DEGs).

### Pathway enrichment analysis

We performed pathway enrichment analysis to determine the biological importance and functional classification of PCOS-specific DEGs. GeneCodis (http://genecodis.cnb.csic.es/) [17–19] and WebGestalt (WEB-based GEne SeT AnaLysis Toolkit) (http://bioinfo.vanderbilt.edu/webgestalt/) [20] web-based enrichment analysis tools were used for this pathway analysis.

### Quantitative real-time PCR (qRT-PCR)

PCOS-specific DEGs between MGCs and CCs were validated with quantitative real-time PCR (qRT-PCR). qRT-PCR analysis was performed on the Roche LightCycler^®^ 480 using gene-specific primers and SYBR Green I Master mix (Roche). Gene-specific primers are listed in S1 Table. The 2^-ΔΔ*C*T^ method [21] was used to analyze the qRT-PCR results with ACTB as a normalization factor.

### Statistical analysis

Unpaired Student’s t-test was used to test the statistical significance of differentially expressed genes between MGCs and CCs for both PCOS and control groups. Paired Student’s t-test was used to test the statistical significance of PCOS-specific DEGS validated by qRT-PCR. p<0.05 was considered to be statistically significant.

PCOS-specific DEGs were clustered using hierarchical clustering and their distance values were calculated using the average linkage method.

## Results

### Correlation of datasets

Applying our search filtering criteria, we found three microarray datasets (GSE10946, GSE34526 and E-MEXP-3641) that had been performed with Affymetrix GeneChip Human Genome U133 Plus 2.0 arrays.

GSE10946 (Kenigsberg *et al*.) [10] contained 23 cumulus samples (12 from PCOS patients, 11 from controls). GSE34526 (Kaur *et al*.) [9] contained 10 granulosa samples (7 from PCOS patients, 3 from controls). E-MEXP-3641 (Grøndahl *et al*.) contained 25 control samples (12 with granulosa cells, 13 with cumulus cells). Thus, the three studies provided a total of 58 samples, comprising 7 granulosa samples, 12 cumulus samples from PCOS patients and 15 granulosa, 24 cumulus samples from controls (Table 1). We also confirmed that all patients in the studies were of reproductive age (between 26–39) and had all received the same IVF treatment.

**Table 1:** Datasets used for meta-analysis.

	PCOS		Control	
	Granulosa	Cumulus	Granulosa	Cumulus
GSE10946	-	12	-	11
GSE34526	7	-	3	-
E-MEXP-3641	-	-	12	13
**Total**	7	12	15	24

The raw data files for the samples were combined and uploaded to the ArrayAnalysis web tool for correlation analysis. The combined datasets were found to be highly correlated (Pearson’s r≥0.8) (S1 Fig).

### Identification of PCOS-specific differentially expressed genes

Raw data were normalized and analyzed using BRB-ArrayTools to identify differentially expressed genes between CCs and MGCs. The two groups were defined as PCOS and control. In the PCOS group, 1,390 genes were differentially expressed in CCs compared to MGCs with a minimum 4-fold change (p<0.001). In the control group, 32 genes for CCs compared to MGCs were also significantly differentially expressed with a minimum 4-fold change (p<0.001). DEG lists for each group are given in S2 Table.

**Fig 2:**
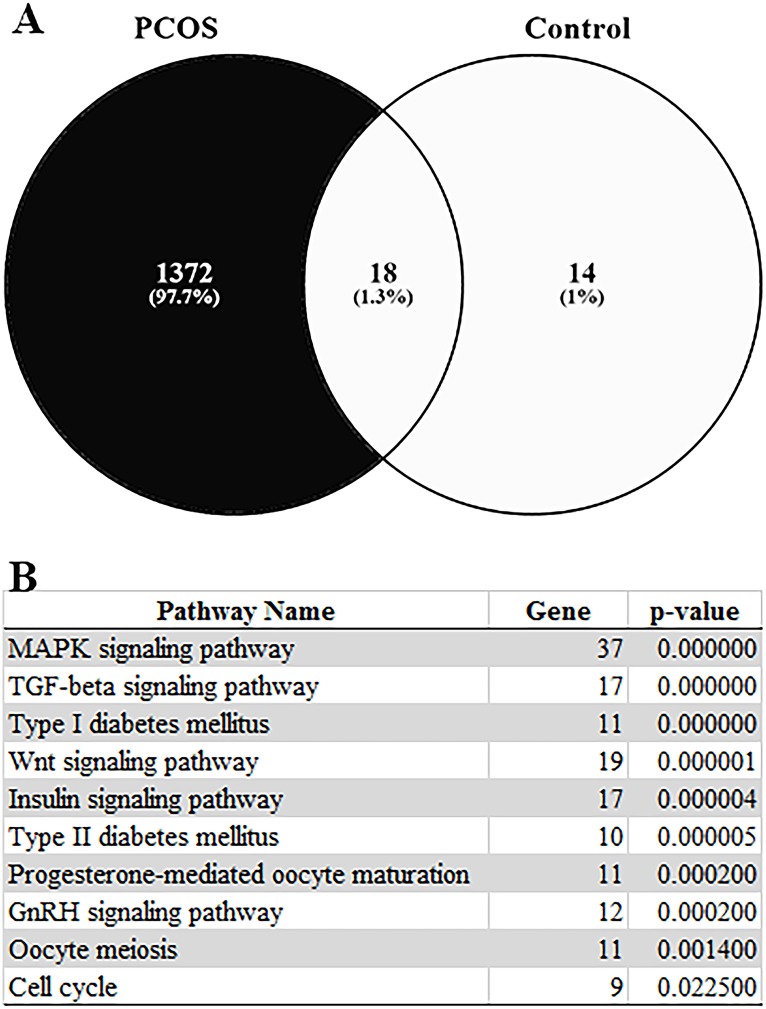
(A) Venn diagram of DEGs for PCOS and control groups obtained from meta-analysis with 1372 genes defined as PCOS specific and 18 genes in common. (B) Pathway enrichment analysis of the 1372 PCOS-specific DEGs.

To identify common DEGs between PCOS and control group, the two datasets were intersected using the Venny Venn’s diagrams drawing tool with 18 genes listed as common DEGs. After eliminating these common DEGs, 1372 genes were identified as PCOS-specific DEGs (Fig 2).

### Pathway enrichment analysis with PCOS-specific DEGs

Pathway enrichment analysis with PCOS-specific DEGs was performed using the WebGestalt pathway analysis toolkit. The KEGG (Kyoto Encyclopedia of Genes and Genomes) [22] pathway enrichment results showed that most PCOS-specific DEGs were significantly enriched (p<0.0225) in PCOS-related pathways like MAPK (Mitogen-Activated Protein Kinase) signaling pathway, TGF-beta signaling pathway, diabetes mellitus related pathways, insulin signaling pathway, oocyte-cell maturation pathways and hormonal signaling pathways (Fig 2).

Since 37 of the PCOS-specific DEGs were enriched in the MAPK signaling pathway (S2 Fig), we specifically focused on those genes that are members of this pathway to perform further analysis.

**Fig 3:**
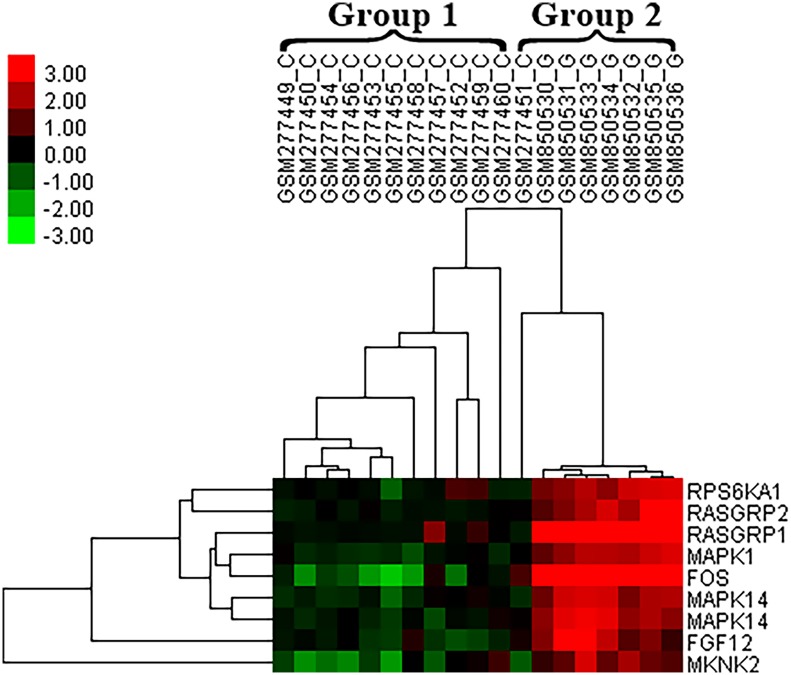
Hierarchical clustering of samples from meta-data and MAPK signaling pathway-related genes, differentially expressed in the meta-analysis study between MGCs and CCs in PCOS group. Cumulus cells were successfully grouped in Group 1, in which expression of MAPK signaling pathway related genes was downregulated.

Hierarchical clustering analysis demonstrated that the expression profiles of some MAPK signaling pathway members, such as MAPK1 (Mitogen-Activated Protein Kinase 1), MAPK14 (p38) (Mitogen-Activated Protein Kinase 14), FGF12 (Fibroblast Growth Factor 12), MKNK2 (MAP Kinase Interacting Serine/Threonine Kinase 2), RPS6KA1 (Ribosomal Protein S6 Kinase, 90kDa, Polypeptide 1), FOS (FBJ Murine Osteosarcoma Viral Oncogene Homolog) and RASGRP1-2 (RAS Guanyl Releasing Protein 1–2), enable CCs to be successfully distinguished from MGCs in PCOS. The expression of these members was also drastically downregulated in group 1, in which CCs are located, whereas they were upregulated in MGCs (Fig 3). On the basis of these results, we further investigated MAPK signaling pathway members (p38 (MAPK14), ERK (MAPK1) and FOS) in independent PCOS samples using qRT-PCR. This confirmed that their expression was downregulated in CCs compared with MGCs.

### Validation of PCOS-specific DEGs by qRT-PCR

Differential transcription levels of PCOS-related genes between CCs and MGCs were tested using qRT-PCR in independent PCOS patients (n = 12) to validate the PCOS-specific DEGs. CCs and MGCs were obtained from the same patients. Eight PCOS-specific DEGs were selected for validation. Four PCOS-related genes and 3 MAPK signaling pathway-related genes (MAPK1, MAPK14 and FOS) were significantly differentially expressed (fc>1.5; p<0.05) between MGCs (n = 12) and CCs (n = 12).

**Fig 4:**
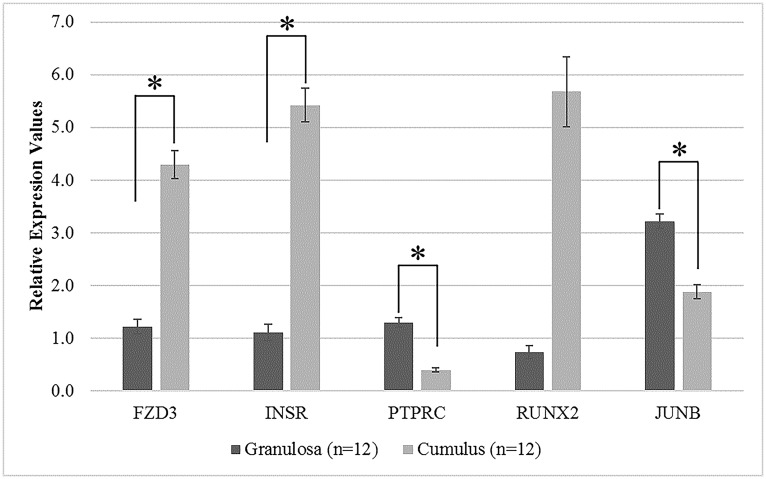
Relative expression of candidate genes (FZD3, INSR, PTPRC, RUNX2 and JUNB) obtained from qRT-PCR analysis. The validation study was performed with 12 independent patients who provided two somatic cell types: CC and MGC. Relative expression values for each gene is shown on the y-axis in arbitrary units. The data are presented as mean ± SEM. Asterisk (*) indicates a significant difference (p<0.05) in gene expression between granulosa and cumulus cells.

Transcript levels of FZD3 (Frizzled class receptor 3) and INSR (Insulin receptor) genes were found to be significantly increased in CCs (3.53-fold and 4.86-fold respectively; p<0.028) while RUNX2 expression increased 7.68-fold in CCs compared with MGCs. Expression of two genes (PTPRC; 3.23-fold and JUNB (Jun B proto-oncogene); 1.71-fold) were significantly (p<0.045) decreased in CCs compared with MGCs (Fig 4).

**Fig 5:**
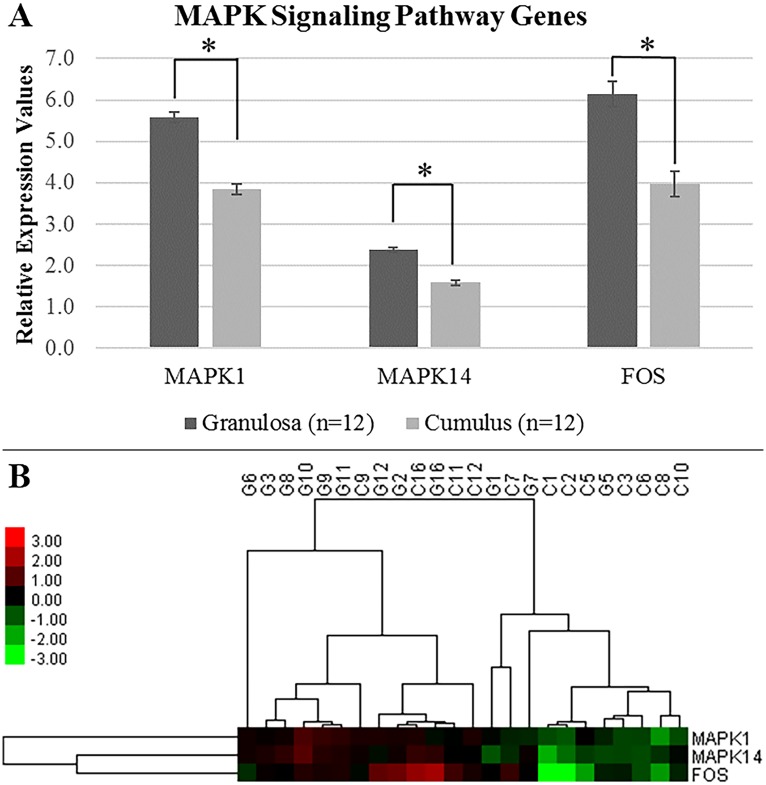
Relative expression values and heatmap figure of MAPK signaling pathway-related genes. (A) Relative expression values obtained from qRT-PCR analysis for each gene are shown on the y-axis in arbitrary units. The data were obtained from 12 independent PCOS patients. Each patient provided both cell types, CC and MGC. The data are presented as mean ± SEM. Asterisk (*) indicates a significant difference (p<0.05) in gene expression between granulosa and cumulus cells. (B) Hierarchical clustering analysis was performed with qRT-PCR data. The CCs (C) and MGCs (G) were grouped according to the expression profiles of three MAPK signaling genes. Their expression levels were lower in CCs than MGCs.

Both the enrichment results and clustering profiles of MAPK signaling-related genes were remarkably significant for PCOS that qRT-PCR validation of MAPK signaling pathway-related genes (MAPK1, MAPK14 and FOS) was performed exclusively with independent PCOS samples (n = 12). The transcription levels of MAPK signaling pathway-related genes were decreased (MAPK1; 1.46- fold, MAPK14; 1.50- fold, FOS; 1.55- fold) significantly (p<0.05) in CCs compared with MGCs (Fig 5).

## Discussion

It is known that granulosa cells (GCs) differentiate into CCs and MGCs during folliculogenesis-oogenesis [5]. As differentiated states of GCs, CCs have different functions, hence different gene expression profiles. In the literature, only one study has compared the gene profiles of MGCs and CCs, and this study only included control samples with normal ovulation functions that were undergoing IVF because of male factor infertility (E-MEXP-3641) [5]. Thus, no previous studies have investigated gene expression differences between these cells in PCOS. Two studies have identified gene expression differences exclusively for MGCs (GSE34526) [9] and CCs (GSE10946) [10] in PCOS and normal samples. The present study was able to identify DEGs between the two cell types specific to PCOS by combining these three studies bioinformatically through meta-analysis. Meta-analysis is a valuable tool for combining raw or processed data from many different, independent studies. While methodological differences, like platform differences, are a common problem for many meta-analyses, we were lucky enough to find sufficient data generated using the same platform (Affymetrix GeneChip HG-U133 Plus 2.0). In addition, after normalization, these datasets were found to be highly correlated (r>0.8), which allowed us to compare them directly. To the best of our knowledge, this is the first meta-analysis study that identifies differentially expressed genes between MGCs and CCs in PCOS.

We identified PCOS-specific DEGs by eliminating those genes that were differentially expressed in the control group between MGCs and CCs. This showed that 32 genes were differentially expressed (fc≥4, p<0.05) between MGCs and CCs in the control group. However, the number of DEGs in the PCOS group increased to 1390, with18 being common with the control group. Thus, the increased number of DEGs in these cell types from PCOS patients may indicate changes in the regulation of molecular mechanisms in the PCOS state compared to control samples.

The pathway enrichment analysis conducted with the 1372 PCOS specific DEGs showed that these genes were specifically enriched in oocyte-follicle maturation and PCOS related pathways, such as MAPK signaling [23], TGFß signaling [24], insulin signaling [25], GnRH signaling [26] and Wnt signaling [27] pathways.

### Wnt signaling and CCs

The Wnt signaling pathway is a developmental pathway that determines cell fate by leading the cell to proliferation or differentiation [28]. There are three types of Wnt signaling pathway: the canonical Wnt pathway, the non-canonical planar cell polarity pathway and the non-canonical Wnt/Ca^2+^ pathway. The three pathways all start activation of the signal by binding WNTs to their specific receptors (FZDs). Frizzled receptors (FZDs) have the N-terminal extracellular cysteine-rich domain (CRD) for binding WNTs as ligands. The canonical Wnt pathway is related to regulation of gene transcription. The key element in this pathway is ß-CATENIN, which is regulated by a regulator complex in the cytoplasm. This complex includes GSK3 (Glycogen synthase kinase 3), APC (Adenomatous polyposis coli), CKIα (Casein kinase I-α) and AXIN. The canonical Wnt/ß-CATENIN pathway is activated by ligands (WNTs) that bind to FZD and its co-receptor LRP6 (low-density lipoprotein receptor-related protein 6) or LRP5 (low-density lipoprotein receptor-related protein 5). After this activation, regulated ß-CATENIN is released from the regulator complex before traveling to the nucleus to activate Wnt-related transcription factors [29]. Our meta-analysis study showed that 19 of PCOS-specific DEGs, including FZD3, FZD5 and LRP5, were enriched in the Wnt signaling pathway and overexpressed, which is important for Wnt signaling activation.

Among the FZDs, FZD3 binds to the ligand WNT2 (Wingless-Type MMTV Integration Site Family Member 2), located at the top of the Wnt signaling pathway [30]. Mammalians studies have revealed the role of Wnt signaling for normal ovarian development [28,31], while its dysregulation was related to PCOS [30]. Moreover, these experiments have shown that Wnt signaling pathway receptors (FZD) have normal expression values in MGCs in control samples [31]. Our meta-analysis showed that FZD3 gene expression is at the same level in CCs and MGCs for control samples. However, according to our qRT-PCR results, FZD3 expression level was increased in CCs 3.53-fold compared to MGCs in PCOS patients. CCs have to be fully differentiated just before ovulation, which is an important step in oocyte development. Thus, the high level of FZD3 in CCs may indicate ongoing differentiation activity, which may be the reason for immature oocyte-follicle development.

### INSR and PCOS

It has been well studied that insulin resistance, diabetes and obesity are closely related with PCOS [32–34]. Concordant with the literature, many of the PCOS-specific DEGs obtained from our meta-analysis were enriched in insulin and glucose-related pathways like Type 1 and 2 diabetes mellitus, and insulin signaling pathways. Until now, studies have mostly focused on one of the common key players in these pathways, INSR and related INSR-specific SNPs, and dysregulation in the expression of this gene in PCOS [35,36]. Additionally, Purcell *et al*. report that INSR is a regulator of insulin-dependent glucose uptake in CCs, which are responsible for providing pyruvate as an energy source to oocytes [37]. In our study, INSR expression was found to be up-regulated (3.03-fold) in CCs compared to MGCs while we also confirmed that INSR expression was up-regulated in CCs using qRT-PCR. One might speculate that increased expression of INSR in CCs may be the reason why more energy is supplied to immature oocytes that are unable to complete their developmental process.

### MAPK signaling pathway, JUNB and maturation

Previous studies have revealed that, following luteinizing hormone and follicle stimulating hormone stimulation in the follicle and COC, activation of MAPK signaling initiates meiotic resumption and CC expansion. It is also known that secretion of oocyte paracrine factors for maturation process is triggered by MAPK signaling activation in CCs [38]. Other studies have shown that MAPK signaling is related to oocyte maturation via ERK1/2 and p38 (MAPK14) activation in CCs [39,40]. Yamashita *et al*. report that, during *in vitro* maturation of porcine COCs, induction of ERK1/2 and p38 expression may cause cumulus growth and oocyte maturation [39]. As shown in Fig 2 and S2 Fig respectively, 37 of the genes that this study found to be differentially expressed in CCs compared to MGCs in PCOS were enriched in the MAPK signaling pathway with most being downregulated. Since activation of the MAPK signaling pathway is closely related to proliferation, differentiation and the cell cycle, it can be proposed that downregulation of key members of the pathway, such as TGF-ß1 and its receptor TGFBR2, p38 (MAPK14), Ras activating protein RasGRP, ERK (MAPK1) and transcription factor c-FOS, may block the pathway or diminish its activity, thereby preventing CC differentiation and oocyte maturation.

In immature GCs, JUNB and FOS (a member of the MAPK signaling pathway) expression levels increase in response to FSH and LH induction. JUNs are related to GCs’ terminal differentiation into luteinized GCs while these transcription factors (JUN/FOS) also play a key role in terminal differentiation of somatic cells and follicle development [41]. Our study validates that JUNB and FOS expression levels are slightly decreased (1.71 and 1.55-fold respectively) in CCs in PCOS. Lower levels of JUNB and FOS expression may be related with the aforementioned blocking of terminal differentiation in cumulus and granulosa cells and the prevention of oocyte maturation.

In conclusion, the present meta-analysis study allows us to suggest that especially the MAPK signaling pathway, and the insulin and WNT signaling pathways are dysregulated pathways between CCs and MGCs in PCOS. Our qRT-PCR analysis performed with independent PCOS patients further validates these results. Taking our meta-analysis results together with the qRT-PCR validations provides evidence that molecular signaling is dysregulated through MGCs, CCs and the oocyte in PCOS, which is important for follicle and oocyte development and may contribute to the pathogenesis of PCOS.

These results will help to further clarify the molecular basis and functional significance of several pathways and which pathway member genes are involved in PCOS pathogenesis.

## Supporting Information

S1 Fig Correlation plot of arrays. 58 samples from 3 studies were found to be highly correlated with each other (r≥0.8).
(TIF)

S2 Fig MAPK signaling pathway. Reprinted from KEGG MAPK Signaling Pathway figure (map04010) [22] under a CC BY license, with permission from KEGG/GenomeNet, original copyright 2016. This figure was obtained from KEGG. Genes, which were labeled with red were found to be down-regulated in CCs and blue were found to be up-regulated in CCs in our study.
(TIF)

S1 Table The list of differentially expressed genes. PCOS-specific DEGs, Common DEGs and Control-specific DEGs were given in 3 independent sheets. Each sheet includes the p-value, fold change and annotation of each DEG.
(XLSX)

S2 Table The list and the sequences of the primers used in the validation study. (XLSX)
