# Supplementary figures and images for: Identification of Polycystic Ovary Syndrome (PCOS) Specific Genes in Cumulus and Mural Granulosa Cells
Source: PLoS One. 2016 Dec 20;11(12):e0168875. doi: 10.1371/journal.pone.0168875 (PMC5173369; doi:10.1371/journal.pone.0168875)

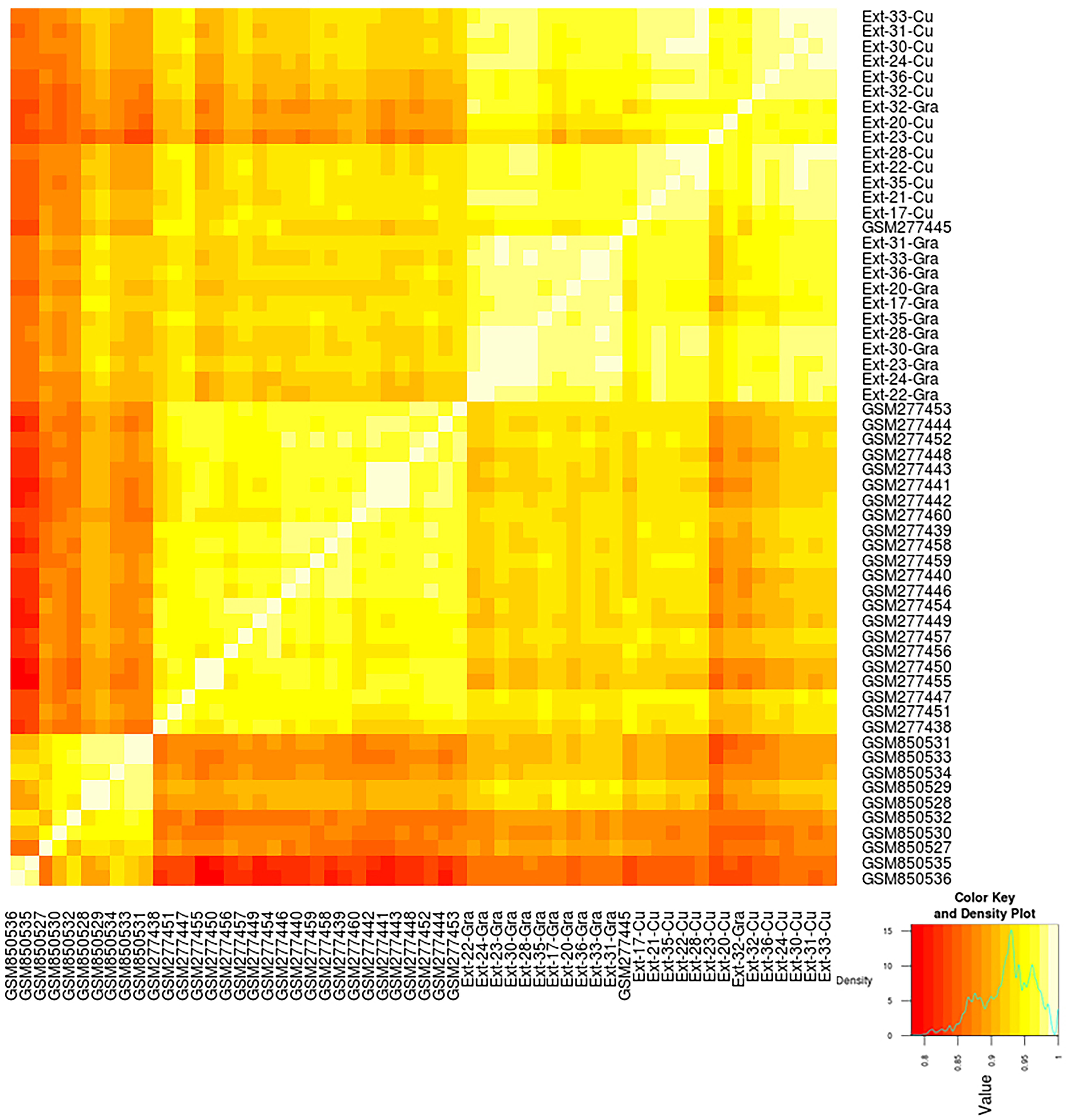

Supplement: S1 Fig — 58 samples from 3 studies were found to be highly correlated with each other (r≥0.8). (TIF) [file pone.0168875.s001.tif]

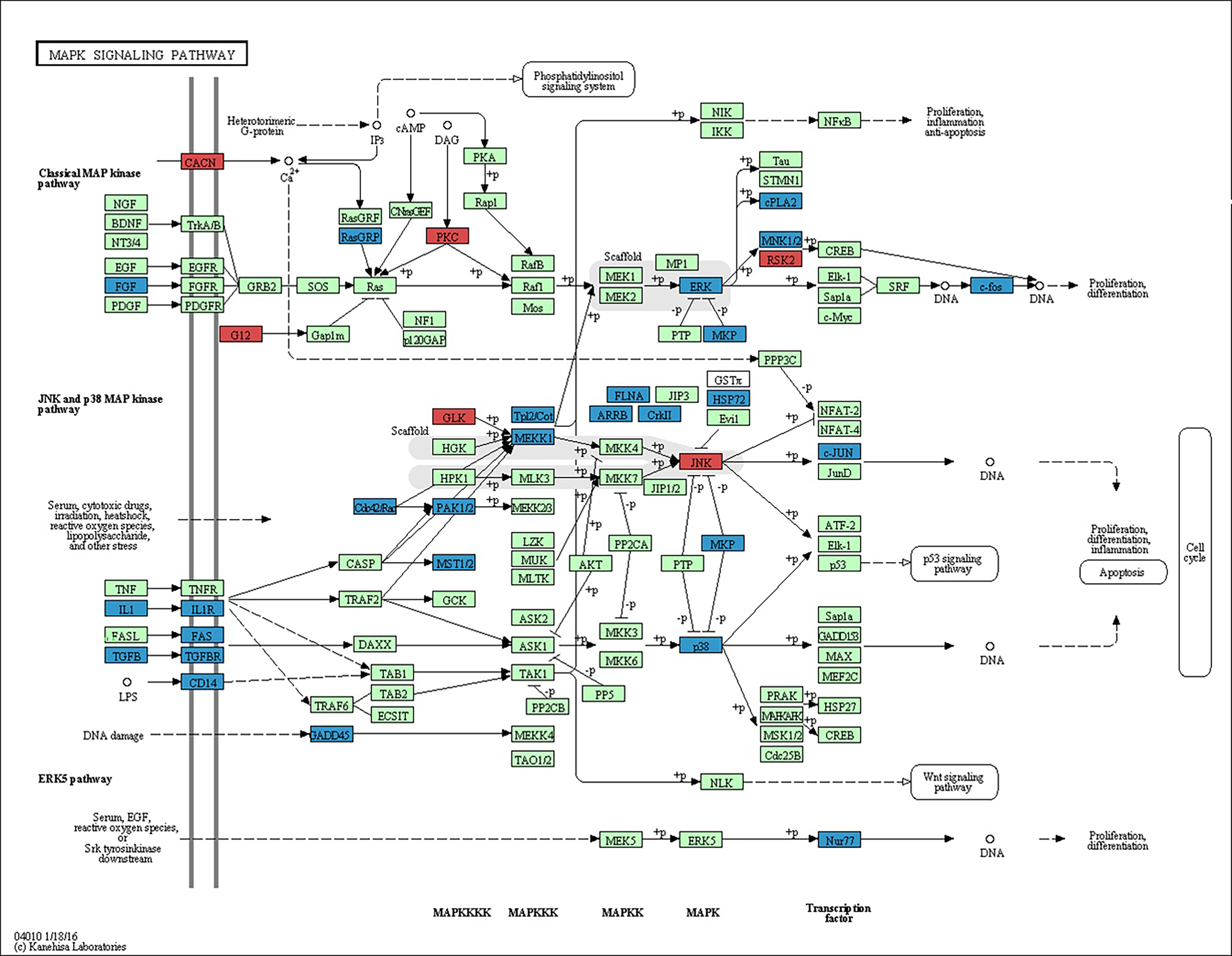

Supplement: S2 Fig — Reprinted from KEGG MAPK Signaling Pathway figure (map04010) [22] under a CC BY license, with permission from KEGG/GenomeNet, original copyright 2016. This figure was obtained from KEGG. Genes, which were labeled with red were found to be down-regulated in CCs and blue were found to be up-regulated in CCs in our study. (TIF) [file pone.0168875.s002.tif]
